# Performance enhancement in the workplace: why and when healthy individuals should disclose their reliance on pharmaceutical cognitive enhancers

**DOI:** 10.3389/fnsys.2015.00013

**Published:** 2015-02-25

**Authors:** Mirko D. Garasic, Andrea Lavazza

**Affiliations:** ^1^The Federmann School of Public Policy and Government Campus, The Hebrew University of JerusalemJerusalem, Israel; ^2^Centro Universitario InternazionaleArezzo, Italy

**Keywords:** pharmaceutical cognitive enhancers, privacy, fairness, autonomy, performance-enhancing substances, moral duty, social duty

## Abstract

The use of pharmaceuticals cognitive enhancers (PCE) has been stirring growing interest, not only in the scientific domain but also in the popular media, and has probably had some increase recently in academic, professional and military quarters. So this phenomenon is deemed as a normal procedure aimed at improving the performance of an individual as well as the overall standards of an organization. Although the vast majority of countries have some kind of restrictions to reduce the wide non-medical usage of PCE, these can be overcome quite easily. In arguing for our explicit claim that, in many contexts, the use of cognitive enhancers should be disclosed—as a moral and socially relevant duty—we maintain that PCE present typical, or at least not rare, properties. The features are the following: (a) the enhancer has acute and/or chronic effects. In the first case, shortly after taking the drug the performance is significantly better than average; in the second case, there is a growing or lasting effect, which, however, is poised to diminish when one stops taking the drug; (b) those effects are significant (there is a difference in the outcome considered between taking and not taking the drug) and sometimes dramatic; and (c) a third feature, not directly related to enhancers as such, is their varying safety, availability, and legal permissibility, which might either induce people to take them or refrain them from doing so. We will consider the issue of fairness due to “unenhanced” people as well as the potentially dysfunctional social consequences of an undisclosed PCE use.

## Introduction

In recent years, the use of pharmaceuticals cognitive enhancers (PCE) has been stirring growing interest, not only in the scientific domain but also in the popular media, and has probably had some increase in academic, professional and military quarters, although such increase is very difficult to assess (McCabe et al., 2005, 2007; Teter et al., 2005; Sahakian and Morein-Zamir, 2007; Russo et al., 2008; Franke et al., 2014). PCE can be tentatively defined as drugs which have been shown to improve to some degree some features of human cognition, namely attention, executive functioning (planning, inhibition, and problem-solving), memory and learning, via altering specific neurotransmitters. The most investigated and probably used PEC are off-label drugs primarily aimed at treating neurodegenerative diseases, ADHD, or narcolepsy, which are taken by the healthy to enhance their performance (Greely et al., 2008; Lanni et al., 2008; Marchant et al., 2009; Advokat and Scheithauer, 2013; Mereu et al., 2013; Sattler et al., 2013; Wood et al., 2013; Urban and Gao, 2014) across university campuses (Babcock and Byrne, 2000; Shillington et al., 2006; DeSantis et al., 2008; here we talk about “academic doping”, see Cakic, 2009) as well as other competitive contexts (Sahakian and Morein-Zamir, 2007; Chandler, 2012). For reason of space, arguments related to the current restrictions in place (Smith and Farah, 2011) concerning the purchase of these “smart drugs” (Mehlman, 2004)^1^—and their very limited impact—(Farah et al., 2004; Herman-Stahl et al., 2007) will not be discussed here. Instead, this paper aims at analyzing a specific problem related to PCE use. In particular, it is our goal to show the tension between the *local effect* (the enhancement of the individual performance) and the *global effects* (the unwanted social results deriving from the spreading of the use of PCE). Those effects are to be related to two distinct concepts, which— in that case—are at odds: (1) autonomy, and consequently privacy, due to people in their choice of enhancing themselves; and (2) fairness, which is a socially appreciated value. In the following sections we shall considered them separately, starting with the former and later considering its implications on the latter.

## Local effect as privacy

In arguing for our explicit claim that the use of cognitive enhancers should be disclosed, we maintain that such enhancers present typical, or at least not rare, properties. The features are the following: (a) the enhancer has acute and/or chronic effects. In the first case, shortly after taking the drug the performance is significantly better than average; in the second case, there is a growing or lasting effect, which, however, is poised to diminish when one stops taking the drug; and (b) those effects are significant (the outcome is different depending on whether the drug was taken or not) and sometimes dramatic; (c) a third feature, not directly related to enhancers as such, is their varying safety, availability, and legal permissibility, which might either induce people to take them or refrain them from doing so. Some recent review studies (Lucke et al., 2011; Smith and Farah, 2011) show that a number of scholars are inclined to say that today’s enhancers present those features in a small percentage. Yet, recent developments in the public acknowledgement of PCE use in workplaces put doubts over the accuracy of these statements.^2^

Since some scholars argue that current enhancers have little effect on cognition, it is helpful to highlight their overall effects. A recent qualitative study conducted by Scott Vrecko showed that Ritalin and Adderall do affect intellectual capacities (such as executive functions, working memory and information process), but they *also* heavily affect the user’s emotional states. “Such alterations appear to be an important dimension of the drug effects that users perceive to enable improved academic performance” (Vrecko, 2013). Participants said that the perception of better emotional or affective states was the most important feature of the enhancers, leading to improvements in the sense of having augmented skills in doing academic work. Emotional dynamics are a salient dimension of the use of stimulant-based medications. Altered emotional states caused by cognitive enhancers are “part of what makes stimulant drugs useful in relation to academic work”. This explains why the specifically cognitive effect appears limited. Instead, Ritalin and Adderall have an important action on the dopaminergic system: they affect the attention, the system of pleasure and that of emotions, including a euphoric effect (Racine and Forlini, 2010; Volkow et al., 2012). Therefore, if, as it seems, enhancers are increasingly spreading among intellectual professionals, it means that the users expect a positive and significant effect, at least at the level of subjective perception. Yet, even though research in the field is at the outset, psychostimulants seem to have very complex, dose and context-dependent effects (Konrad et al., 2004; Wood et al., 2013). The role of age, gender, and ethnic groups in drug efficacy is not clear as well, and such factors play a role in creating differences in neurotransmitter systems of individuals. Thus, the effectivity of PCE is deemed to be unreliable. People relying on PCE for their ordinary or extraordinary performances cannot adequately assess the effects of taking different doses of PCE in different contexts and they would often think they are better than they actually are. And that turns in further reason for disclosing the use of PCE beyond those we expose below.

There are no doubts however, that one of the issues that appear more problematic about the idea of disclosing one’s use of PCE is that of entering the private sphere of one’s (moral) conduct. As Warren and Brandeis famously put it (Warren and Brandeis, 1890), privacy is part of a more general right to immunity of the person and—through the principle of “inviolate personality”—it preserves a space of non-interference by others. The right to privacy seems to suggest that creating publicly embarrassing or accusatory situations against the individual should not be tolerated (Prosser, 1960). The enhancement of individual performances through legal drugs seems to pertain to such a scenario. If we are to accept a “public intrusion” in such a choice of lifestyle, it is thus necessary to provide a counterbalancing moral reason that could legitimize the request for a disclosure of such information. When is it possible to identify, then, a moral duty or a social interest that goes against individual privacy in the case of PCE? We shall look into this question in the next section.

## Global effects as unfairness

Interestingly, indeed, it has been recently suggested that PCE might represent a shortcut to the redistribution of (cognitive) wealth (Kohn, 2014). Starting from another study conducted by Mullainathan and Shafir (2013) (in which they underlined that external inputs related to the participants’ income substantially affect their cognitive response in the experiment), Marek Kohn argues that—while not being an ideal solution—taking a pill to reduce the impact of the external variables related to the contingent economical disparities between individuals might be the way to go. We will focus on fairness in competitive contexts. As we shall see, in general those situations are such that some researchers, workers or students who use PCE can be considered more skilled and better at performing their task than they really are. But this is not the main problem *per se*. The potentially dysfunctional social consequences are due to the undisclosedness of the PCE use. If one does not have her drug dose available, she will not be capable of the same performance as usual and, in some professions, might even put her colleagues at risk. If standards are set based on PCE-using workers, this may lead to an organizational dysfunction. Some brief examples can present realistic scenarios in which the principle of the duty of disclosure seems perfectly suitable and reasonable. These examples will highlight the argument for the moral and socially relevant duty to disclose the use of cognitive enhancers, insofar as it is sufficiently harmful or unfair to third parties, though not sufficiently harmful or unfair to justify a legal or organizational ban.

John and Susan are researchers in the same university department. They are good friends but are now competing to get a new position opening up in the department. The university committee has decided to put them to a test to prepare for which they are given 2 weeks off work. What they do not know is that the committee will be evaluating both the *content* of their answers, and the time they will take to complete the test. John chooses to take a 15-mg Adderall tablet, so as to increase his capacity to concentrate and maximize the efforts of his studies. To be fair to Susan, he offers her the same tablet, but she refuses, claiming that it would be unfair to alter her performance for the sole purpose of passing the test. In the end, John gets the job. Although he gave one correct answer less than Susan, he did so in a much shorter time, and so the committee opted for him: they want knowledge, but *also* efficiency. In discovering this, Susan regards the results as clearly unfair: had she taken Adderall as well—she believes—her reaction time in answering the questions would have been comparable to John’s. In this way, she would have won for the higher number of correct answers. She thus approaches John urging him to publicly acknowledge that he took Adderall before the test. He refuses to do so for two reasons: first, he *did* give her the chance to have “completely equal” starting conditions, it was only *her choice* not to take the tablet. Second, there is no official requirement to acknowledge the use of any legal substance: why did she not state that she had three coffees that morning?^3^ Susan objects that, first, the starting conditions would have been unfair in any case because—unless they decided to take Adderall for the rest of their lives—their performance would still not correspond to their real, normal capacities. Second, coffee is well known by the committee, and they probably assume that people under examination would take some. On the contrary, Adderall’s effects are little known, and they vary for each person.

Now consider Robert. He wishes to help his community and has decided to become a professional nurse in the emergency unit of the local hospital. As he approaches the day of the entrance test, he becomes more and more nervous: he knows that if he could only concentrate fully during the written test, he would have a very good chance to pass. He is told by a friend that Ritalin, a legal drug, would increase his alertness, so he decides to give it a try. On the day of the test, he takes his “smart pill” and enters the room of the written exam. After having performed in a way that he deems very close to perfect, he discovers that the practical test is going to follow in 1 h. Given that he still has a few pills left, he takes another one. Even in this context, he can tell that his performance has improved. In line with what he expected, Robert is accepted as a nurse in the emergency unit. Wanting to “help more”, however, he decides that he will take Ritalin regularly when in service. He does so for 6 months, until he runs out of tablets. This happens just on the evening when the local stadium in town crumples down. At both the psychological and the psychical level, Robert is shaken by this “deprivation” and his colleagues have to help him stay focused a few times during the night: this has never happened before. Luckily the wounded are taken care of properly and in a relatively short time, and Robert’s “under-performance” does not cause any particular damage. However, his boss notices the change and asks him what happened. Having a clean conscience and thinking he had done nothing wrong, Robert tells the truth. His boss is extremely angry at his words: notwithstanding the moral questionability of taking PCE for the tests, the main issue is that he needs to know who can perform what, and under what circumstances. They are dealing with life and death: anything preventing him from having the actual picture of the people working under him potentially jeopardizes the success of their medical assistance. For this reason, Robert’s boss asks him to resign. Robert is shocked and objects that he only thought he was going to be more useful, and certainly he did not perceive this as a “secret”. It simply never came out in a conversation.^4^

With short notice, the Ministry of Education occasionally sample tests high school students. The goal is to assess the quality of education, in order to have quantitative data to make comparisons between different towns and regions, identifying effective methods to improve the performance of both teachers and students where needed. Let us assume that standardized student assessment tests are also submitted to those who are old enough to legally take PCE. Even if the tests are anonymous, situations could arise in which students take PCE to improve their results, perhaps encouraged by the teachers themselves, who are likely to aspire to high rankings for their classes. In this way, certain classes or schools in certain towns and regions may end up having very high scores, which, however, would have nothing to do with the teaching methods used or with the students’ abilities. This would cause evident biases in the interpretation of the results and lead to inefficient decisions (let us not forget that we are dealing with sample tests, and that a handful of classes, picked using statistically valid methods, can be used as indicators for vast geographic areas). On the basis of the analysis of the data collected, school authorities may well think that, in those classes in which students took PCE, the quality of the education was higher due to the teaching methods used, and decide to experiment with these methods elsewhere as well. This would result in a waste of public resources and hinder research on truly better teaching methods. Additionally, it could hide actual differences between geographic areas, resulting in a lack of intervention where school performance is actually poor.

In all the cases considered, there would appear to be both a moral duty and a social interest in reporting the use of PCE. Indeed, the use of PCE could be deemed fully legitimate and in fact, if openly reported, it could become an additional element in the assessment of ways to improve one’s performance. To focus on this last case, by knowing what classes, in what geographic areas, took cognitive enhancers, it will be possible to assess their effectiveness without introducing biases in the overall results. This is certainly of social interest as it helps with the efficient allocation of resources. Yet, there also seems to be a moral duty, since teachers who encouraged their students to take PCE without reporting it might be trying to hide their shortcomings as educators. In other contexts, instead, taking PCE unbeknownst to evaluators may result in the failure to identify situations of economic or cultural distress, because school performance has improved only thanks to the use of cognitive enhancers.

## Local vs. global effects

The duty to disclose the use of cognitive enhancers (and maybe also of mood affecting drugs) is not just intended to prevent *local effects* from prevailing on *global effects*—with undesirable social outcomes deriving from unpredictable composition effects. This is certainly the most important consequentialist reasoning that can justify the duty of disclosure. What is at stake, however, is also an ethical principle of fairness that seems to prevail over the right to privacy as we described it earlier. In fact, those who make use of cognitive enhancers in single occasions or non-repeatable competitive situations seem to be required to report the sudden difference that has arisen in their abilities without this enhancement depending on something they can be credited for. Suppose a candidate for a test, on her way to the university, finds the answer sheet that the examiner has lost from her pocket; or that the computer on which a test is run is defective and shows the correct answers together with the questions; or that, even a single time, one gets help from a renowned scientist in conducting a difficult experiment (but claiming full credit for it); or that a junior broker can rely on the advice of a knowledgeable friend who is well introduced in the sphere of finance.

These are situations in which fairness in the competition is lost without violating any rules or prohibitions, but in which the performance has little to do with the actual skills that a candidate may manifest later and with what he has done to improve them. Therefore, fairness in respecting the equality of the starting conditions as a principle of respect for the others leads to reveal aspects related to chance or to the sporadic and extrinsic improvement of one’s skills. It is a much needed principle, weaker than the prohibition to resort to means extraneous to the competition or the duty to waive potential favorable conditions (the latter principle would severely limit individual autonomy). As our first example shows, if all relevant information is made available, the competition is fair and everyone can choose how to act within the framework of the existing legislation.

The duty to disclose the use of PCE has a final consequence that one should consider. We have talked mainly about a moral duty to disclose, but such a duty could also carry a legal obligation and a number of consequences. For example, would that disclosure justify that a person is not hired for a position? We think that a simple answer is not available. In some cases, the hirer could judge that the person is perfectly suitable for the position and the variability of her performance based also on PCE is always satisfactory. In other cases, if the person’s performance with PCE is near the lower threshold of the minimum performance, considering the dose and context-dependent effects, it could be a risk to rely on her, so the hirer would have good reasons not to hire her. In light of these considerations, there might be a question about the effective incentives (perhaps stressing the moral praiseworthiness of disclosing) to declare the use of PCE. Such incentives deserve attention, but they cannot be analyzed here.

## Conclusion

The aim of this paper—including the use of realistic examples—has been to shed light over the existing (but overlooked) tension between the *local* and *global effects* of the use of PCE. Even if it is perhaps problematic to accept in a liberal society at first, a partial reduction of our right to privacy might be necessary in order to preserve the equally important principle of fairness (as well as social safety and efficiency) that an unregulated use of PCE could threaten. The legitimacy of this step derives from other values deeply entrenched in liberal societies, such as the restriction of individual autonomy if this puts in jeopardy that of others. Reasons related to equity and social interests, therefore, can suggest that there is a moral duty to publicly acknowledge the use of PCE in order to limit the potential damages. There have already been a number of scientific and moral assessments of PCE in the literature, and we do not expect to have provided an exhaustive account on how to legislate further on PCE with this work, but we do hope to have offered some innovative ways of conceptualizing the debate evolving around the increasing use of PCE.

## Conflict of interest statement

The authors declare that the research was conducted in the absence of any commercial or financial relationships that could be construed as a potential conflict of interest.

## Footnotes

^1^Maxwell Mehlman provides a useful account of the use, effects and contexts of these drugs (Mehlman, 2004).

^2^In a recent interview for example, an Australian Public Servant admitted that in May 2014 there was an extensive (and widespread) use of modafinil in order to complete the Federal Budget on time (Farr, 2014).

^3^It should be noted that this argument is often used by bio-liberals: given that caffeine is also a cognitive enhancing substance, why should we limit the use of one enhancer over another? For a more precise analysis see Julien (2001), especially pages 145–164.

^4^A similar motivation could be applied to the renowned cases of cocaine use among surgeons around the world. However, our intention is to focus on PCE precisely because—unlike cocaine—they are *not* illegal drugs.
